# Draft Genome Sequence of DLB, a *Dyella*-Like Bacterium from the Planthopper *Hyalesthes obsoletus*

**DOI:** 10.1128/genomeA.00686-16

**Published:** 2016-07-21

**Authors:** Tamar Lahav, Einat Zchori-Fein, Vered Naor, Shiri Freilich, Lilach Iasur-Kruh

**Affiliations:** a Department of Natural Resources, Newe Ya‘ar Research Center, Agricultural Research Organization, Ramat Yishay, Israel; b Department of Entomology, Newe Ya’ar Research Center, Agricultural Research Organization, Ramat Yishay Israel; c Department of Plant Sciences, Golan Research Institute, Katzrin, Israel; d Ohalo College, Katzrin, Israel; e Department of Biotechnology, ORT Braude College, Karmiel, Israel

## Abstract

We report here the draft genome sequence of a *Dyella*-like bacterium (DLB) isolated from *Hyalesthes obsoletus*, the insect vector of the uncultivable mollicute bacterium “*Candidatus* Phytoplasma.” This isolate inhibits *Spiroplasma melliferum*, a cultivable mollicute. The draft genome of DLB consists of 4,196,214 bp, with a 68.6% G+C content, and 3,757 genes were predicted.

## GENOME ANNOUNCEMENT

*Dyella*-like bacterium (DLB) is a Gram-negative, aerobic, rod-shaped bacterium that belongs to the family *Xanthomonadaceae*. It was isolated from the planthopper *Hyalesthes obsoletus* (Hemiptera: Cixiidae), an insect vector of the uncultivable mollicute bacterium “*Candidatus* Phytoplasma,” which causes yellows disease in grapevine (1). DLB inhibits *in vitro* the cultivable mollicute *Spiroplasma melliferum* (2).

DLB was grown in a liquid medium containing 6 g/liter Luria broth, 2 g/liter K_2_HPO_4_, and 0.5 g/liter KH_2_PO_4_ (pH 7) at 28°C and 150 rpm for 48 h. Bacteria were collected by centrifuge (3,500 × g; 15 min) and washed twice with phosphate-buffered saline (PBS) (8 g/liter NaCl, 0.2 g/liter KCl, 1.44 g/liter Na_2_HPO_4_, and 0.24 g/liter KH_2_PO_4_ [pH 7.5]). The genomic DNA of a DLB pellet was isolated using the QIAamp DNA minikit (Qiagen, Germany), according to the manufacturer’s protocol. The quality of DNA was examined using the NanoDrop spectrophotometer (Thermo Scientific). The genome sequencing of DLB was performed at the DNA Services Facility (Chicago, IL); a mate-pair library using the Nextera mate-pair sample preparation kit was generated, followed by genome sequencing in Illumina MiSeq, using 2 × 100 reads. *De novo* assembly was done using the software package CLC Genomics Workbench (version 7.0) after quality trimming of raw sequence data of <Q20, which yielded 6,709,847 reads from 333 contigs, with approximately 86.7-fold coverage. The *N*_50_ quality measurement of the contigs was 42.2 kb, with an average contig size of 12.5 kb, and the largest contig assembled was approximately 191.2 kb. The genome contains 4,191471 bp, with a G+C content of 68.6%.

Gene prediction analysis and functional annotations were performed within the Integrated Microbial Genomes-Expert Review (IMG-ER) platform (http://img.jgi.doe.gov) developed by the Joint Genomics Institute (JGI) (3), using the Department of Energy (DOE)-JGI Microbial Genome Annotation Pipeline. A total of 3,832 genes were predicted, including 3,757 coding sequence (CDS) predictions using Prodigal version 2.50 (4), 49 tRNA genes using tRNAscan-SE 1.3.1 (5), and 5 rRNA genes using HMMER 3.0 (6). The predicted coding sequences were translated and used to search the TIGRFam, Pfam, KEGG, COG, and InterPro databases. A total of 1,945 protein-coding genes were assigned to 1,381 KO categories.

To provide an initial assessment of candidate compounds that may serve as *Phytoplasma* inhibitors, gene clusters associated with the biosynthesis of secondary metabolites were identified by the antiSMASH pipeline (7). A total of five potential clusters for secondary metabolites were identified in the DLB genome, including three bacteriocin clusters, one cluster of terpene, and one cluster of nonribosomal peptides (NRPs).

### Nucleotide sequence accession number.

This genome shotgun sequence has been deposited at GenBank under the accession no. LFQR00000000.
